# Fruit Physiology through Signaling Processes: Latest Advances and Future Challenges

**DOI:** 10.3390/ijms24020976

**Published:** 2023-01-04

**Authors:** Luciano Freschi, Francisco J. Corpas, José M. Palma

**Affiliations:** 1 Departamento de Botânica, Instituto de Biociências, Universidade de São Paulo, Sao Paulo 05508-090, Brazil; 2 Group of Antioxidants, Free Radicals and Nitric Oxide in Biotechnology, Food and Agriculture, Estación Experimental del Zaidín, Spanish National Research Council (CSIC), C/Profesor Albareda 1, 18008 Granada, Spain

Fruits are unique to flowering plants and confer a selective advantage to these species by facilitating seed maturation and dispersal. In fleshy fruits, intense mitotic activity occurs after pollination and fruit set. This is followed by a gradual reduction in cell division rate, maximal rates of cell enlargement, and a subsequent gain of competence to initiate ripening (Figure 1). The biodiversity of flowering plants is reflected in multiple patterns of fruit growth and physiology across different species, including variations in the metabolic rewiring associated with ripening.

The ripening of fleshy fruits is usually associated with structural, biochemical, and physiological changes, including modifications in the general appearance, texture, flavor, and aroma, which ultimately convert immature fruits into considerably more attractive and palatable structures, making them more attractive to seed dispersal animals [1]. Therefore, fruit ripening represents a key reproductive strategy that maximizes the efficacy of seed dispersal, and thus facilitates the growth and success of the next generation. Moreover, from a human perspective, fruits are critically important horticultural products. They provide a significant source of nutrients, aromas, and taste, and sustain a multibillion dollar global market focused on fresh fruits and fruit derivatives (e.g., juices, pastes, food additives).

Intricate signaling networks orchestrate every step, from fruit set to fruit ripening and senescence. With this perspective in mind, this Special Issue on Fruit Physiology through Signaling Processes: Latest Advances and Future Challenges was organized. This volume provides new insights, ranging from the hormonal regulation of fruit development and the identification of potential therapeutic uses of fleshy fruits, to alternative ways to extend fruit shelf-life, among other emerging findings in this area.

Briegas et al., (2020) [2] provide a bonafide example of the complex regulatory pathways controlling ripening-associated events by combining differential gene expression analysis and hormonal profiling in the abscission zone (AZ) and pericarp tissues of olive (*Olea europaea* L.) fruits. Jasmonic acid (JA) was significantly more abundant in the AZ than in pericarp tissues at the ripe stage, which is when olive fruit abscission takes place. These transcriptomic data allowed for the characterization of unique and common transcriptional signatures related to cell wall modification; plant hormone metabolism and signaling; vesicle trafficking; and ion fluxes between the fruit tissues. This offers new insight into the hormonal and metabolic regulation processes that are potentially involved in the cell wall modifications that occur during fruit abscission.

Cell wall modifications are also intrinsically associated with the reduction in firmness of fleshy fruits, triggered during ripening. Additionally, the actions of specific signaling molecules, particularly ethylene, have long been considered to control enzyme-mediated cell wall disassembly. Adding to this knowledge, Molinett et al. (2021) [3] revealed that the gas signaling molecule hydrogen sulfide (H_2_S) also participates in the control of pectin and hemicellulose catabolism, as well as the expression of genes encoding for key cell wall-modifying enzymes during the postharvest period of strawberry (*Fragaria chiloensis*) fruits. Moreover, it was found that treatment with optimal doses of H_2_S significantly extended the shelf-life, delayed the rapid decay, softening, and loss of fruit peel lightness typically observed in strawberry fruits undergoing senescence. 

As a consequence of the metabolic rewiring of ripening fruits, chemical substances, including the so-called nutraceutical compounds, are differently accumulated depending on the ripening stage and the conditions encountered by the fruit during its ripening. In a comprehensive metabolomic analysis of sweet pepper (*Capsicum annuum* L.) fruits at two ripening stages, under control and nitric oxide (NO)-enriched atmosphere, Guevara et al., (2021) [4] identified 12 bioactive compounds which differentially accumulated, depending on the ripening stage and the treatment with NO. Some of these nutraceutical compounds, including capsoside A (one of the capsaicinoids associated with pepper pungency), quercetin and derivatives, and gingerglycolipid A were predominantly found in green pepper fruits. In contrast, phytosphynosin, L-tryptophan, tetrahydropentoxylin, and blumenol C glucoside were present in higher levels in red fruits. In addition to promoting an increase in pepper ascorbate content of about 40% [5], NO treatment was also shown to promote the accumulation of many other bioactive compounds, including flavonoids (quercetin and derivatives), gingerglycolipid A, L-tryptophan, and blumenol C glucoside. This research presents the characterization of ripening and NO-regulated changes occurring in multiple substances present in pepper fruits. These substances are potentially used by humans for consumption and economic benefit; thus, these findings introduce a myriad of possibilities for biotechnological improvements in this important crop species.

In addition, Wang et al. (2020) [6] provided an extensive review of the expression patterns and roles played by aquaporins during plant vegetative and reproductive development. Among various other functions, aquaporin-mediated water uptake, into the large vacuoles of fleshy fruit cells, is highlighted as a critical aspect that influences fruit growth dynamics. As summarized by the authors, many aquaporin-encoding genes are highly expressed at the fruit-expanding stage; however, examples of aquaporin genes being expressed throughout fruit development, or even upregulated during ripening, are not uncommon. This collection also provides insight into the regulatory mechanisms controlling seed germination by the glycolytic enzyme ENO2 [7] and tuber dormancy through the cysteine-rich peptide Snakin-2 [8]. These regulatory mechanisms, alongside fruit biology, are critically relevant to the developmental processes that occur in the contexts of agriculture and food production.

## Figures and Tables

**Figure 1:**
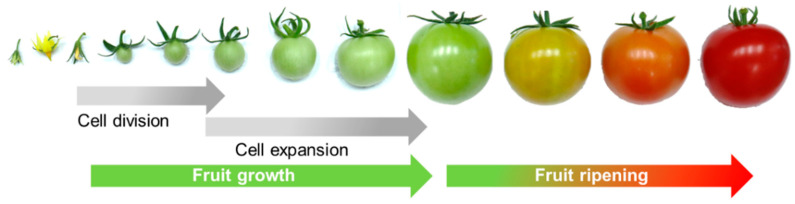
Main stages of tomato (*Solanum lycopersicum* L.) fruit development, exemplifying the two main phases of fruit growth (cell division and cell expansion) followed by the initiation and progression of ripening.
